# Synthesis of an aqueous, air-stable, superconducting 1T′-WS_2_ monolayer ink

**DOI:** 10.1126/sciadv.add6167

**Published:** 2023-03-22

**Authors:** Xiaoyu Song, Ratnadwip Singha, Guangming Cheng, Yao-Wen Yeh, Franziska Kamm, Jason F. Khoury, Brianna L. Hoff, Joseph W. Stiles, Florian Pielnhofer, Philip E. Batson, Nan Yao, Leslie M. Schoop

**Affiliations:** ^1^ Department of Chemistry, Princeton University, Princeton, NJ 08544, USA.; ^2^ Princeton Institute for Science and Technology of Materials, Princeton, NJ 08544, USA.; ^3^ Department of Physics and Astronomy, Rutgers University, Piscataway, NJ 08854, USA.; ^4^ Institute of Inorganic Chemistry, University of Regensburg, D-93040 Regensburg, Germany.

## Abstract

Liquid-phase chemical exfoliation can achieve industry-scale production of two-dimensional (2D) materials for a wide range of applications. However, many 2D materials with potential applications in quantum technologies often fail to leave the laboratory setting because of their air sensitivity and depreciation of physical performance after chemical processing. We report a simple chemical exfoliation method to create a stable, aqueous, surfactant-free, superconducting ink containing phase-pure 1T′-WS_2_ monolayers that are isostructural to the air-sensitive topological insulator 1T′-WTe_2_. The printed film is metallic at room temperature and superconducting below 7.3 kelvin, shows strong anisotropic unconventional superconducting behavior with an in-plane and out-of-plane upper critical magnetic field of 30.1 and 5.3 tesla, and is stable at ambient conditions for at least 30 days. Our results show that chemical processing can make nontrivial 2D materials that were formerly only studied in laboratories commercially accessible.

## Graphical abstract

**Figure:**
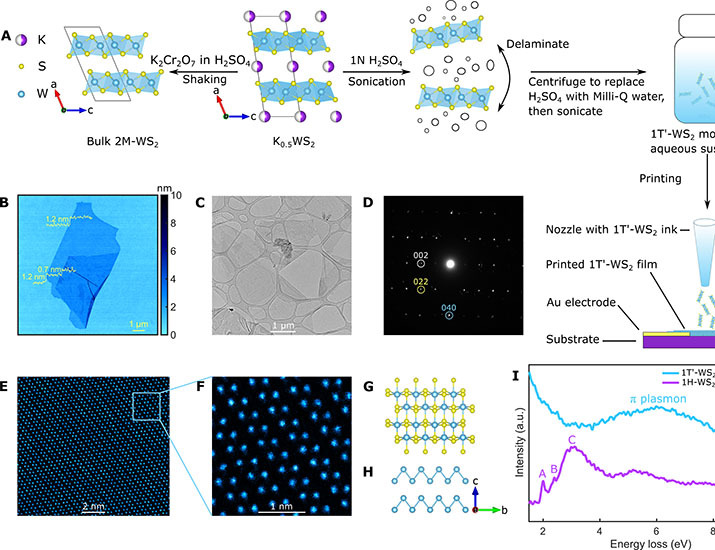

## Teaser

Chemically exfoliated 1T'-WS2 monolayers are processed into an aqueous, air-stable ink, and its printed films are superconducting.

## INTRODUCTION

**Fig. 1.**
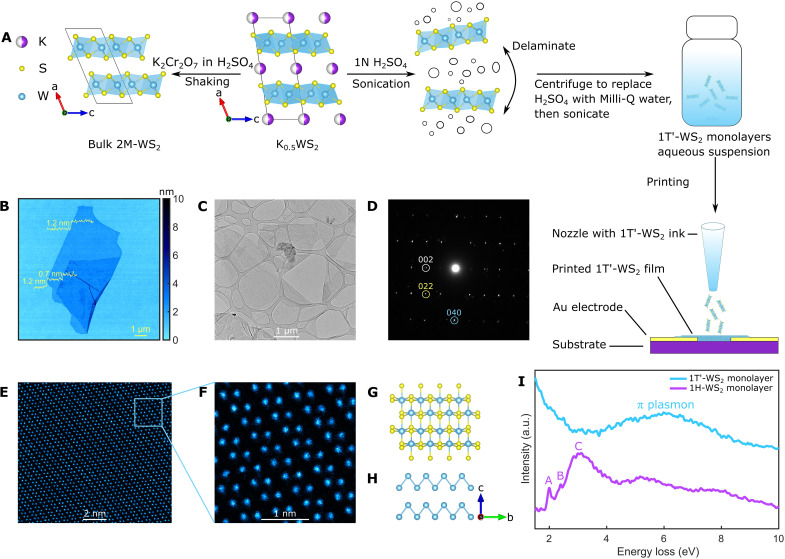
Chemically exfoliated 1T′-WS_2_ monolayers. (**A**) A schematic explaining the synthesis of bulk 2M-WS_2_ made of layered face-sharing distorted WS_6_ octahedra, as well as an aqueous 1T′-WS_2_ nanosheet ink from K_0.5_WS_2_. (**B**) An atomic force microscopy (AFM) image of a monolayer 1T′-WS_2_. (**C**) A transmission electron microscopy (TEM) image of 1T′-WS_2_ monolayers. (**D**) Selected area electron diffraction (SAED) of a 1T′-WS_2_ monolayer. (**E**) An atomic resolution scanning TEM (STEM) image of a monolayer 1T′-WS_2_, where W atoms are highlighted with blue color. (**F**) A zoom-in atomic resolution image of a 1T′-WS_2_ monolayer. (**G**) In-plane crystal structure of monolayer 1T′-WS_2_. The zigzag chains of W atoms are shown in (**H**). (**I**) Electron energy loss spectroscopy (EELS) of a 1T′-WS_2_ monolayer in comparison with a 1H-WS_2_ monolayer. a.u., arbitrary units.

Two-dimensional (2D) materials offer opportunities for the discovery of previously unknownphysics and advanced applications, such as in flexible or wearable electronics (*1*–*6*) (*7*, *8*). One exciting material is monolayer WTe_2_, which is a 2D topological insulator (TI) with an excitonic insulator ground state that becomes superconducting upon gating (*9*–*11*). Monolayer 1T′-WS_2_ had been predicted to be a 2D TI with a larger gap as compared to monolayer WTe_2_ (*12*), but its synthesis is challenging, as WS_2_ prefers to adopt the more stable semiconducting 2H phase (fig. S1, A and B), which can be directly exfoliated into semiconducting 1H-WS_2_ monolayers (fig. S1, C and D) by direct sonication in various organic solvents (*13*). Recently, a new phase of WS_2_ was reported, which brought the synthesis of 1T′-WS_2_ monolayers one step closer; by oxidizing K_0.7_WS_2_ with either K_2_Cr_2_O_7_ in diluted H_2_SO_4_ or I_2_ in acetonitrile, a superconducting 2M-WS_2_ phase (fig. S1, E and F), which consists of 1T′-WS_2_ layers with face-sharing distorted WS_6_ octahedra (fig. S1, G and H) in a two-layer unit cell, can be synthesized (Fig. 1A) (*14*, *15*). This 2M-WS_2_ phase has the highest superconducting transition temperature (*T_c_*) among the transition metal dichalcogenides (TMDs). In a monolayer, the potential combination of superconductivity and topology opens up a route to access non-Abelian states that are key for topological quantum computing (*16*).

2M-WS_2_ can be mechanically exfoliated down to the monolayer limit in its structural 1T′-WS_2_ unit. The mechanically exfoliated 1T′-WS_2_ monolayer has been reported to be metallic; its resistivity drops at 5.7 K but does not reach zero (*15*). Thus, it is unclear whether the monolayer is superconducting.

Chemical exfoliation offers another route toward monolayers, with the advantage that it accesses large quantities, which can then be processed into printable inks, moving studies from the laboratory setting to potential industrial applications, especially if the synthesized ink is stable in air. It is well established that metallic WS_2_ monolayer nanosheets can be synthesized via Li intercalation of 2H-WS_2_ and subsequent sonication in water, but these nanosheets are never purely of the 1T′ phase and usually have many defects (*17*–*22*). While this metallic WS_2_ has been studied extensively for catalytic applications (*20*, *23*), to the best of our knowledge, its superconductivity has never been investigated. In general, high-quality 2D superconducting monolayer suspensions are scarce. Chemical-exfoliated restacked TaS_2_ nanosheets are superconducting with a *T_c_* of 3 K (*24*). 1T′-MoS_2_ nanosheets show a *T_c_* of 4.6 K (*25*), and recently reported printed, electrochemically exfoliated NbSe_2_ nanosheet films have a *T_c_* of 6.8 K (*6*). Of these, only the last material has been successfully used as a printable ink; however, protective organic molecules are necessary to stabilize the ink, as NbSe_2_ is relatively air sensitive (*26*). Furthermore, the ink was synthesized electrochemically, a method limited to metals (*6*), which would exclude 2H-WS_2_ as a starting material.

In this study, we report a simple chemical exfoliation method to make a stable superconducting ink containing primarily monolayers of single-phase 1T′-WS_2_. We show that the sheets are stable in water, which provides a cheap, nontoxic, and abundant ink solvent for potential printable superconducting electronics. Exfoliation with high yield is then achieved by sonication, resulting in a suspension composed of monolayers with lateral sizes up to tens of micrometers, which crystallize in the 1T′ structure. The composition and structure of the products are characterized with multiple diffraction, microscopy, and spectroscopy techniques, establishing that the structure remains intact and low in defects, suggesting that they are of much higher quality than their mechanical or Li intercalation–exfoliated counterparts. We prove that a thin-film cast from the nanosheet ink is superconducting below 7.3 K, with an in-plane upper critical magnetic field of 30.1 T and an out-of-plane upper critical magnetic field of 5.3 T. The film shows highly anisotropic superconducting properties that resemble those observed in gated 1T′-WTe_2_, pointing to 2D superconductivity and a potential exotic origin (*10*, *11*). After exposing the printed film to ambient conditions for 30 days, its electronic transport behavior, as well as its Raman and x-ray photoelectron spectroscopy (XPS) spectra, remain unchanged. Last, we show that, besides water, the exfoliated 1T′-WS_2_ monolayers can be well dispersed in several common solvents such as ethanol, isopropanol (IPA), and dimethylformamide (DMF). The ink forms room-temperature conducting films on various known substrates, such as SiO_2_/Si wafers, borosilicate glass, and indium tin oxide (ITO)-coated glass, as well as flexible substrates such as polyethylene terephthalate (PET), polyethylene naphthalate (PEN), and silicone elastomer. Thus, the 1T′-WS_2_ monolayer ink that we present here has a wide application range, such as 3D printing, integrated circuits, and flexible devices.

## RESULTS

### Chemical exfoliation of 1T′-WS_2_ monolayers

The starting compound K_0.5_WS_2_ was synthesized via a solid-state reaction, with full experimental details reported in the Materials and Methods. To the best of our knowledge, its crystal structure has not yet been reported. We resolved its structure by single-crystal x-ray diffraction (SCXRD; table S1), as shown in Fig. 1A. K_0.5_WS_2_ crystallizes in the monoclinic space group *C*2/*m* and consists of layers of distorted WS_6_ octahedra that are structurally similar to the layers in 1T′ (or T_d_)-WTe_2_ (*27*, *28*). The K atoms in the interlayer space are disordered, and energy-dispersive x-ray spectroscopy (EDS) analysis shows that there are 0.5 K per formula unit (fig. S2).

The parent compound K_0.5_WS_2_ reacts violently upon immersing in the diluted H_2_SO_4_ and releases H_2_ bubbles. In addition, we observed that K intercalated samples show a large increase in interlayer spacing compared to the deintercalated samples. The layer distance increases further if K is only partially deintercalated, visible in the powder XRD (PXRD; fig. S3A). As evidenced by thermal gravimetric analysis (TGA) (fig. S3B), the presence of residual K between the layers accompanies the presence of crystal water, which likely solvates the remaining K between the layers and thus explains the enlarged interlayer spacing. This increased interlayer distance, combined with the force of the evolving H_2_ bubbles and sonication, likely aids exfoliation. Using this insight, we designed a route to chemically exfoliate K_0.5_WS_2_ to 1T′-WS_2_ monolayers by directly sonicating the parent crystals K_0.5_WS_2_ in diluted acid (Fig. 1A). Details of the process can be found in Materials and Methods. A colloidal-stable nanosheet ink in Milli-Q water with a yield of about 20% can be obtained if large unexfoliated pieces are removed via centrifugation at 2000 rpm as indicated by a large, negative zeta potential of −57.5±4 mV (*29*). The negatively charged nanosheets presented here are electrostatically stabilized and form a suspension in water without the extra steric hindrance or charges provided by organic surfactant molecules whose presence is known to hinder the application of the as-synthesized nanomaterials for electronic purposes (*30*).

### Structural characterization of 1T′-WS_2_ monolayers

The diluted nanosheet suspension was deposited on a silicon wafer, and the sheets were characterized with atomic force microscopy (AFM). As shown in Fig. 1B and fig. S4 (A and B), the exfoliated 1T′-WS_2_ nanosheets have a thickness of about 0.7 nm if measured on top of another nanosheet, which agrees well with the monolayer thickness of 1T′-WS_2_. The nanosheet is 1.2 nm thin if measured on the wafer directly, which is commonly reported for chemically exfoliated TMD monolayers on wafers due to absorbed water molecules (*31*–*34*). On the basis of a statistical analysis of more than 200 1T′-WS_2_ nanosheets dispersed on a wafer, we found that the vast majority of the exfoliated 1T′-WS_2_ nanosheets are monolayers (fig. S4C). The median lateral size of the monolayers is about 1 μm (fig. S4D), but larger monolayers with lateral sizes up to 15 μm can also be found easily. Figure 1C shows a typical transmission electron microscopy (TEM) image of 1T′-WS_2_ monolayers randomly stacked on top of each other. The selected area electron diffraction (SAED) on a monolayer 1T′-WS_2_ nanosheet (fig. S5) is shown in Fig. 1D, confirming its high crystallinity and the 1T′ structure (fig. S6). A few additional diffraction peaks are visible, which arise from the fact that several 1T′-WS_2_ monolayers usually lie on top of each other. This can be seen in fig. S5, which shows the image from which the diffraction pattern was taken. An atomic resolution scanning TEM (STEM) image of a monolayer 1T′-WS_2_ is shown in Fig. 1E, with no visible defects and impurity phases. We have analyzed more than 50 nanosheets with local characterization techniques, such as SAED and high-resolution STEM, and found all sheets to be highly crystalline. Figure 1F shows the zigzag chains of W atoms of a typical 1T′-TMD structure (Fig. 1, G and H). S atoms cannot be resolved in Fig. 1F, as STEM imaging is a Z-contrast technique, and S has a much smaller atomic number than W. We confirm the existence of S and its relative ratio to W in the monolayers by EDS (fig. S7). Our AFM and TEM analysis found that the ink seems to be composed of primarily monolayers and that all larger unexfoliated pieces could be successfully removed with centrifugation, as mentioned above. Last, to differentiate these monolayers from their semiconducting 1H counterparts, we performed electron energy loss spectroscopy (EELS) studies on a monolayer 1T′-WS_2_ and a monolayer 1H-WS_2_. The valence EELS spectra are shown in Fig. 1I. In the case of 1H-WS_2_, features appear around 2, 2.4, and 3 eV, which are the A, B, and C excitons that are associated with the electronic properties of the semiconducting phase (*35*–*38*), and the broad peak around 8 eV, which corresponds to the π plasmon (*38*–*40*). In contrast, the single-layer 1T′-WS_2_ does not exhibit exciton features observed in the 1H phase; instead, only one broad peak around 6 eV can likely be attributed to a π plasmon. This clearly distinguishes the electronic properties of 1T′-WS_2_ from its semiconducting 1H counterpart.

### Structure and unconventional superconductivity of the printed 1T′-WS_2_ film

**Fig. 2.**
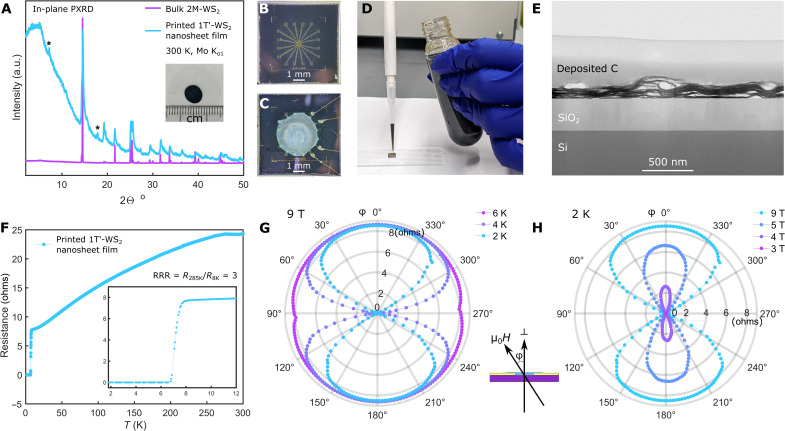
Film printing with the 1T′-WS2 nanosheet ink. (**A**) The 1T′-WS_2_ nanosheet ink was deposited on a polymer film and dried (inset) for an in-plane PXRD characterization with a PXRD machine that works in transmission geometry. The nanosheet PXRD pattern is compared to the in-plane pattern of bulk 2M-WS_2_. The stars indicate additional peaks due to sheets restacking and the contribution from out-of-plane diffraction. (B) A SiO_2_/Si wafer with preprinted electrodes. (**C**) 1T′-WS_2_ nanosheet film deposited on the SiO_2_/Si wafer shown in (B). (**D**) A pipette can be used to deposit the nanosheet ink. (**E**) A bright-field STEM image showing the cross-sectional structure of the printed 1T′-WS_2_ nanosheet film on a SiO_2_/Si wafer. (**F**) Resistance versus temperature data from 300 to 1.8 K for a freshly printed 1T′-WS_2_ film without any external magnetic field. The inset shows the superconducting transition region. RRR, residual resistivity ratio. (**G**) Angle-dependent resistance data of the printed 1T′-WS_2_ device measured from 2 to 6 K with a 9 T external magnetic field when the field is rotated from perpendicular to parallel to the device plane. (**H**) Angle dependence of the resistance measured at 2 K with a 3 to 9T external magnetic field. Inset: An illustration showing the experimental configuration where the magnetic field is perpendicular to the printed device plane at φ = 0° and φ = 180°.

Having established that we can produce an ink made of metallic 1T′-WS_2_ monolayers, we can now study its properties. The ink was first deposited and dried on a polymer film, as shown in the inset of Fig. 2A. The structure of the dried film was characterized by in-plane PXRD in transmission mode. A 2M-WS_2_ crystal was measured in the same way for comparison. As shown in Fig. 2A, the patterns align, suggesting that the sheets retained good crystallinity. Two broad peaks also appear in the pattern of the ink (labeled with stars); these come from some out-of-plane contribution of crumbled sheets in the printed film (*41*–*43*). The Raman spectrum of the printed 1T′-WS_2_ films has the characteristic peaks of the bulk 2M-WS_2_, with extra peaks showing up at 196 and 400 cm^−1^, which can be attributed to the loss of symmetry in the monolayers (fig. S8).

To study the electronic transport properties of the 1T′-WS_2_ nanosheet ink, a droplet was deposited on a silicon wafer with prepatterned electrodes, as shown in Fig. 2 (B to D). The ink droplet was dried in ambient conditions before Au wires were attached to the exposed prepatterned electrodes, as shown in Fig. 2C. To gain an insight into how the nanosheets deposit on the wafer, we cut a sample of a dried nanosheet film on a silicon wafer with a focused ion beam (FIB) and studied its cross section with STEM. A typical bright-field image is shown in Fig. 2E. The film shows that the sheets are in good contact and that the nanosheets randomly stack on top of each other. Although some areas of the sheets are crumbled, the majority of the sheets are well oriented.

The temperature (*T*)–dependent resistance (*R*) in Fig. 2F shows that the device is metallic, as the resistance decreases with decreasing temperature. At ∼7.7 K, the resistance drops sharply and reaches zero at ∼6.6 K (Fig. 2F, inset). We define the *T_c_* as the temperature where the resistance drops to 50% of the normal state resistance, which is 7.3 K. The *T_c_* of the printed 1T′-WS_2_ film is higher than the *T_c_* of the mechanically exfoliated monolayer (*15*) but smaller than that reported for bulk 2M-WS_2_ (*14*, *15*). A similar trend is also reported for NbSe_2_ (*6*, *44*, *45*), where the bulk’s *T_c_* is higher than that of an exfoliated thin film, while the monolayer has the lowest *T_c_*. As the film is 2D in nature, a strong anisotropy with respect to an applied magnetic field (μ_0_*H*) direction can be expected. The angle-dependent resistance under an applied magnetic field of 9 T at different temperatures is shown in Fig. 2G. Similarly, the angle-dependent resistance at 2 K with different magnetic field strengths is shown in Fig. 2H. The external magnetic field is applied perpendicular to the device plane at ϕ = 0° and 180° (out of plane, μ_0_*H*^⊥^) and is parallel to the device plane at ϕ = 90° and 270° (in plane, μ_0_*H*^‖^) [inset between Fig. 2 (G and H)]. The electronic transport is highly anisotropic in the superconducting state; it is more easily suppressed when μ_0_*H*^⊥^ and more robust when μ_0_*H*^‖^ (Fig. 2, G and H). The angle-dependent resistance data shown in Fig. 2, G and H suggest that the resistance signal stems predominately from well-oriented nanosheets despite some crumbling seen in the cross-sectional image (Fig. 2E).

**Fig. 3.**
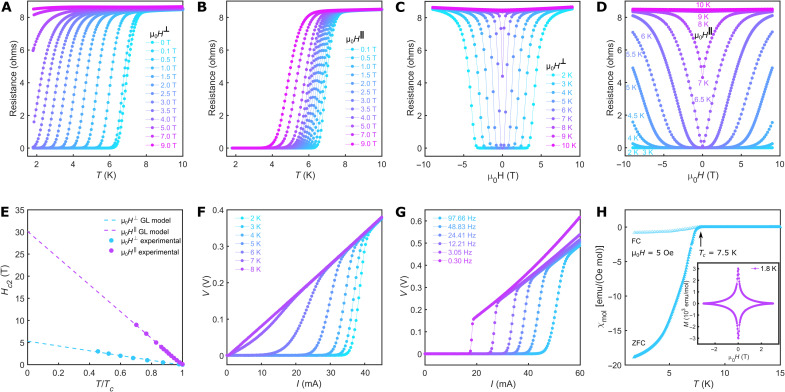
Superconducting properties of the printed 1T′-WS_2_ film. (**A** and **B**) Temperature-dependent resistance (*R-T*) of the printed 1T′-WS_2_ film measured under external magnetic fields ranging from 0 to 9 T, which is applied perpendicular (A) or parallel (B) to the device plane. (**C** and **D**) Isotherms of the printed 1T′-WS_2_ film from 2 to 10 K measured with an external magnetic field that is applied perpendicular (C) or parallel (D) to the device plane. (**E**) Upper critical field *H*_*c*2_ versus *T_c_* plot for both μ*_0_H*^⊥^ and μ*_0_H*^‖^. The experimental data are fitted using the Ginzburg-Landau (GL) theory. (**F**) Current (*I*) versus voltage (*V*) curves of the printed 1T′-WS_2_ film measured from 2 to 8 K with an ac current of frequency 24.41 Hz. (**G**) *I-V* curves measured at 2 K with different ac current frequencies. (**H**) Temperature-dependent magnetic susceptibility of dried 1T′-WS_2_ nanosheet powder collected from the ink. Inset: Magnetic field–dependent magnetization of the same sample measured at 1.8 K.

*R-T* curves at different applied magnetic fields, both with μ_0_*H*^⊥^ and μ_0_*H*^‖^, are shown in Fig. 3 (A and B). The resistance still drops to zero at the highest magnetic field of 9 T if it is applied along the in-plane direction. Figure 3 (C and D) shows the out-of-plane (*R* − μ_0_*H*^⊥^ isotherms) and in-plane (*R* − μ_0_*H*^‖^ isotherms) field-dependent resistance of the printed 1T′-WS2 film around the transition temperature. Below *T_c_*, the *R* − μ_0_*H*^⊥^ isotherms (Fig. 3C) show a broad transition from the superconducting state to the normal state, and their corresponding critical magnetic field decreases as the temperature increases. When μ_0_*H* is applied parallel to the device plane, below 3 K, the resistance remains zero when the applied magnetic field increases to 9 T, suggesting a very high critical magnetic field when applied parallel to the film. To determine the upper critical magnetic field (*H*_*c*2_), the transition temperature at each applied magnetic field, corresponding to half of its normal state resistance, is plotted versus the field for both μ_0_*H*^⊥^ and μ_0_*H*^‖^ (Fig. 3E). A linear correlation of *H*_*c*2_ versus *T_c_* can be modeled by the 2D Ginzburg-Landau (GL) theory (*6*, *15*) for both directions

$$H_{c2}(T)=\frac{{\text{Φ}}_{0}}{2\pi \xi_{\mathrm{GL}}^{2}(0)}\left(1-\frac{T}{T_{c}}\right)$$

where Φ is the magnetic flux quantum and the ξ_GL_(0) is the zero-temperature GL in-plane coherence length. This results in an out-of-plane upper critical magnetic field $\left[H_{c2}^{⊥}(0)\right]$ of 5.3 T and an in-plane GL superconducting coherence length of ξ_GL_(0) ∼7.9 nm. Fitting the in-plane *H*_*c*2_ versus *T_c_* yields an in-plane upper critical magnetic field $\left[H_{c2}^{\|}(0)\right]$ of 30.1 T. Similar to the recently reported mechanically exfoliated few-layer 1T′-WS_2_ (*46*) and printed NbSe_2_ film (*6*), the $H_{c2}^{\|}(0)$ of the printed 1T′-WS_2_ film is very high, far beyond its Bardeen-Schrieffer-Cooper Pauli paramagnetic limit of 13.1 T (*H_p_* ∼1.84 *T_c_*) (*47*). However, the symmetry of the centrosymmetric 1T′ structure of WS_2_ is fundamentally different from the noncentrosymmetric hexagonal structure of NbSe_2_, where Ising-type superconductivity is responsible for exceeding the Pauli limit (*6*, *45*). A similar anisotropy of the critical field, where the in-plane critical field exceeds the Pauli limit, has been observed in 1T′-WTe_2_. It has been pointed out that these unconventional *H*_*c*2_ features can originate from spin-orbit parity (*48*).

The current (*I*) versus voltage (*V*) curves of the device, measured with a fixed ac frequency (24.41 Hz), are shown in Fig. 3F for different temperatures. A critical current (*I_c_*) of ∼33 mA can be extracted at 2 K. The critical current decreases with increasing temperature, and the supercurrent eventually disappears at temperatures above *T_c_*. When the frequency is varied, as shown in Fig. 3G, the critical current of the printed 1T′-WS_2_ film also changes. At 2 K, *I_c_* reaches a maximum of ∼44 mA with an excitation current frequency of 97.66 Hz. On the other hand, it decreases to ∼17 mA with the lowest excitation current frequency of 0.30 Hz. The *I-V* curves become nonlinear above *I_c_* for all the frequencies as Joule heating appears.

A second device (device 2) was printed with a different batch of 1T′-WS_2_ nanosheet ink to confirm the robustness of the cast superconducting films. The electronic transport data of device 2 are nearly identical, and details are shown in the Supplementary Materials (fig. S9).

Next, we studied the dc magnetic susceptibility of a restacked nanosheet pellet that was collected from the dried ink (Fig. 3H). A strong diamagnetic signal is observed below *T_c_* = 7.5 K under zero-field cooled (ZFC) condition. Field cooling (FC) with an applied magnetic field of 5 Oe suppresses the diamagnetic response due to the Meissner-Ochsenfeld effect. The χ_mol_ of the nanosheet pellet at 2 K is within the same order of magnitude as bulk 2M-WS_2_ (fig. S10); thus, the majority of the restacked sample is superconducting. Both the electronic and the magnetic characterizations show that the ink is composed of high-quality superconducting 1T′-WS_2_ nanosheets that are ready to be used for printing electronics on various substrates.

### Phase and air stability

**Fig. 4.**
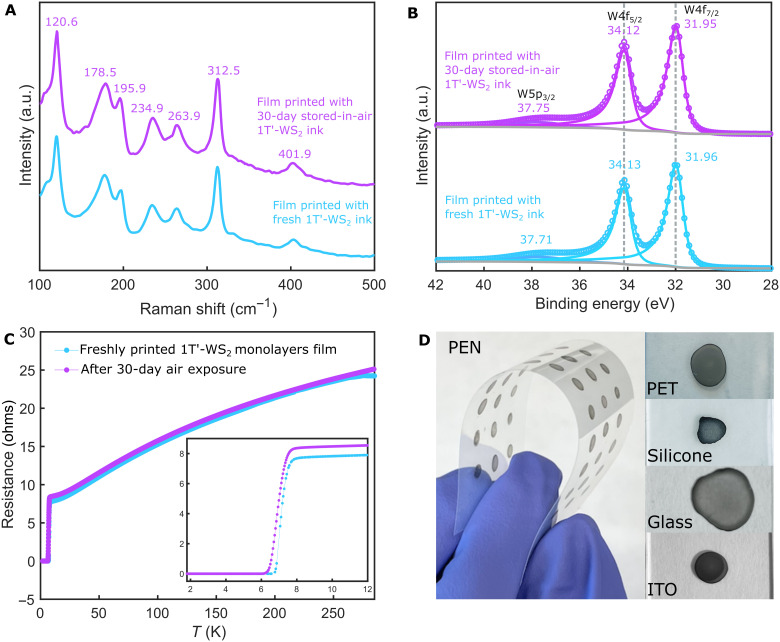
Air stability of 1T′-WS_2_. Raman spectra (**A**) and W XPS spectra (**B**) of the films printed with newly synthesized 1T′-WS_2_ nanosheet ink and the 1T′-WS_2_ nanosheet ink that is stored in air for a month. (**C**) *R-T* curves of the printed 1T′-WS_2_ film measured right after preparing the device and after storing in the air for a month. (**D**) Patterns printed with the 1T′-WS_2_ nanosheet ink on a PEN film, PET, silicone, glass, and ITO. No obvious cracks or fallen-off pieces of the printed patterns are observed while folding the PEN substrate (left), suggesting a good affinity of the 1T′-WS_2_ nanosheet to the flexible substrate.

To investigate the phase stability and air stability of the 1T′-WS_2_ nanosheet ink and the printed device, we stored the ink and both devices in ambient conditions for a month. A new nanosheet film was printed from the air-exposed ink, and its Raman spectrum is identical to that of the freshly printed film (Fig. 4A). Neither the E_2*g*_ nor the A_1*g*_ peaks of the 2H-WS_2_ phase, which should appear at 350.7 and 420.5 cm^−1^, respectively (*15*), are observed in the Raman spectrum, suggesting that the 1T′-WS_2_ nanosheets presented in this study have good phase stability while being stored as a suspension at room temperature. The W and S XPS spectra of both films are also identical (Fig. 4B and fig. S11). The W spectrum is fitted with one set of W4f doublets with W4f_7/2_ at 31.95 eV and W4f_5/2_ at 34.12 eV, respectively, proving that the nanosheets are purely in the 1T′ phase (fig. S12). A full survey spectrum of the printed 1T′-WS2 film on a Cu tape is given in fig. S13. There are no peaks observed at 32.9 or 35.8 eV that would indicate oxidized W species (WO_2_ and WO_3_) (*49*, *50*), suggesting that the 1T′-WS2 nanosheet suspension is air-stable and will not be oxidized when stored at ambient conditions. As 1T′-WS_2_ nanosheets have been reported to be reliable catalysts for hydrogen evolution reaction (HER) (*20*), this finding is expected, as the conditions in HER are harsh, i.e., a voltage is applied to the sheets that are immersed in an aqueous acidic solution. Temperature-dependent resistance measurements on device 1 after 1 month of air exposure show that the device has almost the same room-temperature resistance and the same residual resistivity ratio (RRR = R_285K_/R_8K_) ∼3, and the *T_c_* is almost unchanged as compared to the fresh sample (Fig. 4C). This is different compared to most other known 2D materials that require preparation in an inert environment and the protection of organic molecules to be handled in air (*6*, *51*–*54*). The 1T′-WS_2_ nanosheet ink presented here is robust in water and is stable in ambient conditions without protection, which gives this ink a higher potential for real-world applications.

### Dispersity in different solvents and printability on various substrates

Last, we tested whether the ink can be created with solvents other than water and which substrates the ink can be cast on. Common solvents such as hexane, methanol, ethanol, IPA, acetone, acetonitrile, DMF, tetrahydrofuran, and dimethyl sulfoxide were used to disperse the sheets (fig. S14). The nanosheets can be dispersed in ethanol, IPA, and DMF. Among all tested solvents, water gives the best dispersity and highest stability of the 1T′-WS_2_ nanosheets. The aqueous ink was deposited on various substrates, including hard substrates such as SiO_2_/Si wafers, borosilicate glass, and ITO-coated glass, as well as flexible substrates such as PET, PEN, and silicone elastomer (Fig. 4D). The room-temperature conductivity of the printed nanosheets patterned on different substrates was measured with a multimeter, showing that each film is metallic at room temperature. Thus, the 1T′-WS_2_ monolayer ink can be prepared with various solvents and can be printed on many different substrates, expanding its possible applications to integrated circuits, and flexible devices.

## DISCUSSION

In conclusion, we successfully synthesized monolayers of the 2D TI candidate 1T′-WS_2_ and prepared an air-stable aqueous superconducting ink consisting primarily of 1T′-WS_2_ monolayers. A printed 1T′-WS_2_ film is metallic at room temperature and superconducting below 7.3 K with a maximal critical current of 44 mA at 2 K. The upper critical magnetic field is 30.1 T if the field is applied in plane and 5.3 T in the perpendicular field direction, pointing to unconventional superconductivity. Both the 1T′-WS_2_ monolayer ink and the printed film are stable at ambient conditions for at least 30 days without any protective agents. This also suggests that monolayer 1T′-WS_2_ is air stable and superconducting, opening up avenues for investigating the interplay between topology and superconductivity in this 2D material. We further show that the ink can form conducting films on various substrates, such as SiO_2_/Si wafer, borosilicate glass, ITO, PET, PEN, and silicone elastomer. The simple synthesis, stability, and versatility of the ink reported here suggest that it might find applications in several areas, such as quantum computing, integrated circuits, and flexible and wearable devices. Furthermore, the air-stable monolayers can be studied for their potential interplay of topology and superconductivity.

## MATERIALS AND METHODS

### Chemicals

Potassium sulfide powder (K_2_S, anhydrous, minimum 95%) was purchased from Strem Chemicals. Tungsten powder (W; Puratronic 99.999%) was purchased from Alfa Aesar. Sulfur powder (S; 99.98%) and potassium dichromate (K_2_Cr_2_O_7_; 99.98%) were purchased from Sigma-Aldrich. Sulfuric acid solution (0.5 mol/L) was purchased from LabChem. ITO-coated square glass slides (surface resistivity: 70 to 100 ohms per square) were purchased from Sigma-Aldrich. PET sheets (2 mm, copolymer), PEN films (0.05 mm, biaxially oriented), and silicone elastomer sheets [(C_2_H_6_OSi)n] were purchased from Goodfellow Cambridge Limited. Micro cover glass was purchased from VWR. Milli-Q water, obtained from a Milli-Q purification system (Millipore Sigma), was used in all experiments. 2H-WS_2_ {nanopowder, 90-nm average particle size [scanning electron microscopy (SEM)], 99% trace metals basis} was purchased from Sigma-Aldrich. All the chemicals were used directly without further purification.

### Synthesis of K_x_WS_2_ crystals

K_2_S powder (96.0 mg), 320.2 mg of W powder, and 83.8 mg of S powder were mixed and ground with an agate mortar and pestle in an argon-filled glove box. Then, the mixture was placed into an alumina crucible (LSP Ceramics) before being sealed in a fused silica ampule (inside diameter, 14 mm and outer diameter, 16 mm; Technical Glass Products) under vacuum. The sealed ampule was heated in a furnace to 850°C in 10 hours and maintained at 850°C for 24 hours before slowly cooling down to 550°C at a rate of 3°C/hour. Then, the furnace was turned off to let the ampule cool down to room temperature.

### Synthesis of 2M-WS_2_ crystals with K_2_Cr_2_O_7_ in diluted H_2_SO_4_

This synthesis was repeated as reported earlier (*14*).

### Chemical exfoliation of 1T′-WS_2_ monolayers

To exfoliate 1T′-WS_2_, the as-synthesized K*_x_*WS_2_ crystals were sonicated in 0.5 M H_2_SO_4_ solution in a 1 mg:2 ml solid-to-liquid ratio directly with a Branson 1800 sonicator at low-power mode for an hour. After sonication, the upper liquid was transferred to a centrifuge tube for centrifugation at a speed of 12,000 rpm at 4°C for 30 min to replace the solvent with Milli-Q water. This procedure was repeated twice to remove all the acid. Then, the suspension was sonicated at low-power mode with a Branson 1800 sonicator for another hour before being centrifuged at 2000 rpm at 4°C for 30 min. The supernatant containing thin nanosheets was collected for further characterization, while the sediments containing partially or unexfoliated residue were discarded. For the magnetic measurements, this upper nanosheets suspension was further centrifuged at a speed of 13,000 rpm at 4°C for 30 min for the nanosheets to restack in the bottom of the centrifuge tube. The restacked sheets were dried under vacuum at room temperature for the magnetic measurements.

Plastic spatulas, plastic tweezers, and ceramic blades were used throughout the experiments. No stainless steel–made tools were used in the synthesis to avoid potential contamination.

### Characterization

In-plane PXRD data were collected on a STOE STADI P PXRD with Mo *K*_α1_ radiation and a single Mythen detector, working in transmission geometry. SCXRD was performed on a small crystal picked from the as-synthesized K*_x_*WS_2_ crystals. Measurements were performed at 299(1) K on a Bruker Kappa APEX-II CCD diffractometer with Mo K_α_ radiation (λ = 0.71073 Å). A multiscan absorption correction was applied using SADABS. The data were solved using SHELXT with intrinsic phasing and refined with SHELXL via the least squares method (*55*).

SEM images and EDS spectra were taken with a Verios 460 Extreme High-Resolution Scanning Electron Microscope with an Oxford energy dispersive x-ray spectrometer. The nanosheets were characterized with a Bruker Dimension ICON3 AFM operating in soft tapping mode and Talos F200X S/TEM. The acquired AFM images were processed with Gwyddion software. Atomic resolution STEM images of the nanosheets were collected with the Titan Cubed Themis 300 double Cs-corrected S/TEM, operated at 300 kV with a point resolution of 0.07 nm and an energy resolution of 0.8 eV, which were used for direct imaging and chemical composition analysis of the samples. The Titan Cubed Themis S/TEM is equipped with a Super-X EDS system for elemental mapping and a Gatan Quantum SE/963 P post-column energy filter for energy-filtered TEM data acquisition. The zeta potential of nanosheet suspension was measured with a Mobius (Wyatt Technology) zeta potential detector. The cross-sectional sample of the printed 1T′-WS_2_ film on the SiO_2_/Si device was prepared with a Helios NanoLab G3 UC DualBeam system (FIB/SEM), followed by imaging by the Titan Cubed Themis S/TEM. The EELS acquisition was carried out using a Nion UltraSTEM microscope operated at 60 kV with the convergence and collection semi-angles set at 35 and 16.5 mrad, respectively. The energy resolution of the spectra presented here is 40 meV as estimated from the full width at half maximum of the measured zero-loss peaks. To compare the spectral features of 1T′- and 1H-WS_2_, their zero-loss peaks and the detector response function are deconvoluted from the raw spectra via Fourier-log deconvolution (*56*). The 1H-WS2 used for the EELS study was made by grinding the 2H-WS_2_ nanopowders in acetone and dispersing on a TEM grid.

Electronic transport measurements were performed using the ac transport option of a physical property measurement system (PPMS; Quantum Design). The electrical contacts were made in four-probe geometry using silver paste and gold wire. The magnetic measurements were performed using the vibrating sample magnetometer option of the PPMS (Quantum Design).

The Raman measurements were performed on a Horiba Raman spectrometer with a 633-nm laser source and a neutral-density (ND) filter of 0.6. The XPS measurements were performed on a Thermo Fisher Scientific K-Alpha + x-ray photoelectron spectrometer (XPS/UPS). Nanosheet films with diameters of about 1 cm were printed directly on the Cu tape for the XPS measurement. An x-ray beam with a size of 400 μm was used to measure the XPS spectra of the ensembled 1T′-WS_2_ nanosheets presented in Fig. 4B and figs. S11 and S12. An x-ray beam with a size of 100 μm was used to measure the survey spectrum shown in fig. S13. All the peaks were calibrated according to the C 1s peak at 248.5 eV (*15*). TGA was performed under air flow with a PerkinElmer TGA 8000 thermogravimetric analyzer.

### DFT calculations

Quantum chemical calculations were performed in the framework of density functional theory using a linear combination of Gaussian-type function scheme as implemented in CRYSTAL17 (*57*, *58*). Calculations were performed for WS_2_ and monolayers of WS_2_. Grimmes D3 dispersion correction was added to full structural optimizations with the generalized gradient approximation (GGA) Perdew-Burke-Ernzerhof (PBE) xc functional (*59*–*62*). The basis sets were taken from literature (*63*, *64*). For W, the diffuse d-shell was removed. The convergence criterion considering the energy was set to 1 · 10^−8^ arbitrary units with a k-mesh sampling of 8 × 8. Vibration frequencies including Raman intensities were computed on the basis of the relaxed structures using the coupled-perturbed Kohn-Sham mode (*65*, *66*). The modes were visualized and animated with the J-ICE application (*67*).
