# Supplementary material for: Synthesis of an aqueous, air-stable, superconducting 1T′-WS2 monolayer ink
Source: Sci Adv. 2023 Mar 22;9(12):eadd6167. doi: 10.1126/sciadv.add6167 (PMC10032609; doi:10.1126/sciadv.add6167)
Supplement: Supplementary file 1 — Figs. S1 to S14 Table S1 [file sciadv.add6167_sm.pdf]

Science Advances
AAAS

Supplementary Materials for

Synthesis of an aqueous, air-stable, superconducting 1T′-WS_2 monolayer ink

Xiaoyu Song *et al.*

Corresponding author: Leslie M. Schoop, lschoop@princeton.edu

Sci. Adv. **9**, eadd6167 (2023)
DOI: 10.1126/sciadv.add6167

This PDF file includes:

Figs. S1 to S14
Table S1

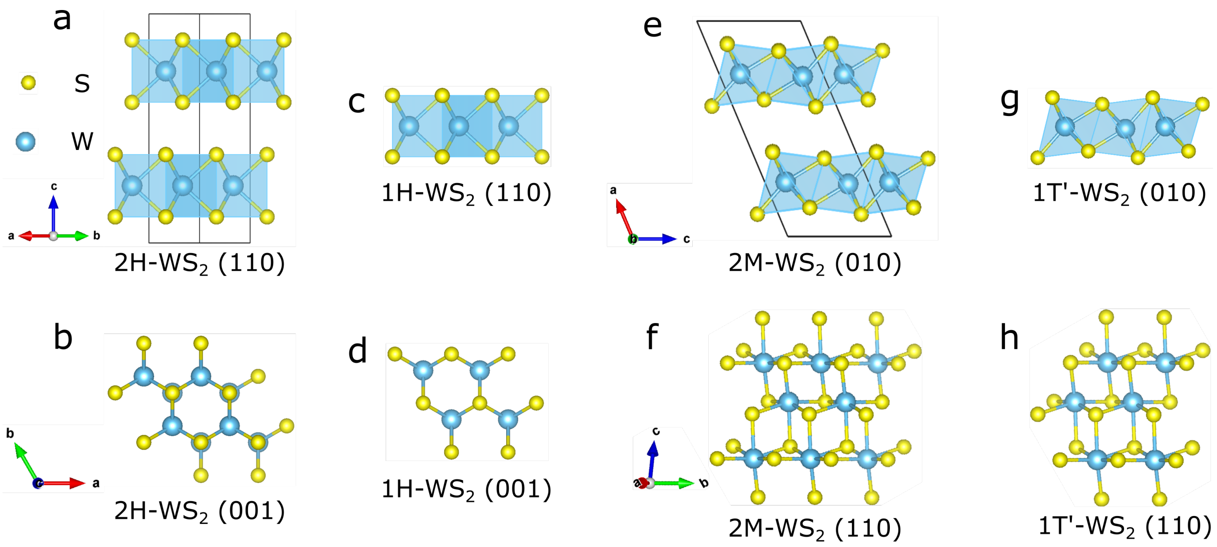

Fig. S1.: **Crystal structures of bulk 2H-WS_2, monolayer 1H-WS_2, bulk 2M-WS_2, and monolayer 1T'-WS_2 from side view and projection view.** (a) Side view of bulk 2H-WS_2; (b) In-plane projection of bulk 2H-WS_2; (c) Side view of monolayer 1H-WS_2; (d) In-plane projection of monolayer 1H-WS_2; (e) Side view of bulk 2M-WS_2; (f) In-plane projection of bulk 2M-WS_2; (g) Side view of monolayer 1T'-WS_2; (h) In-plane projection of monolayer 1T'-WS_2.

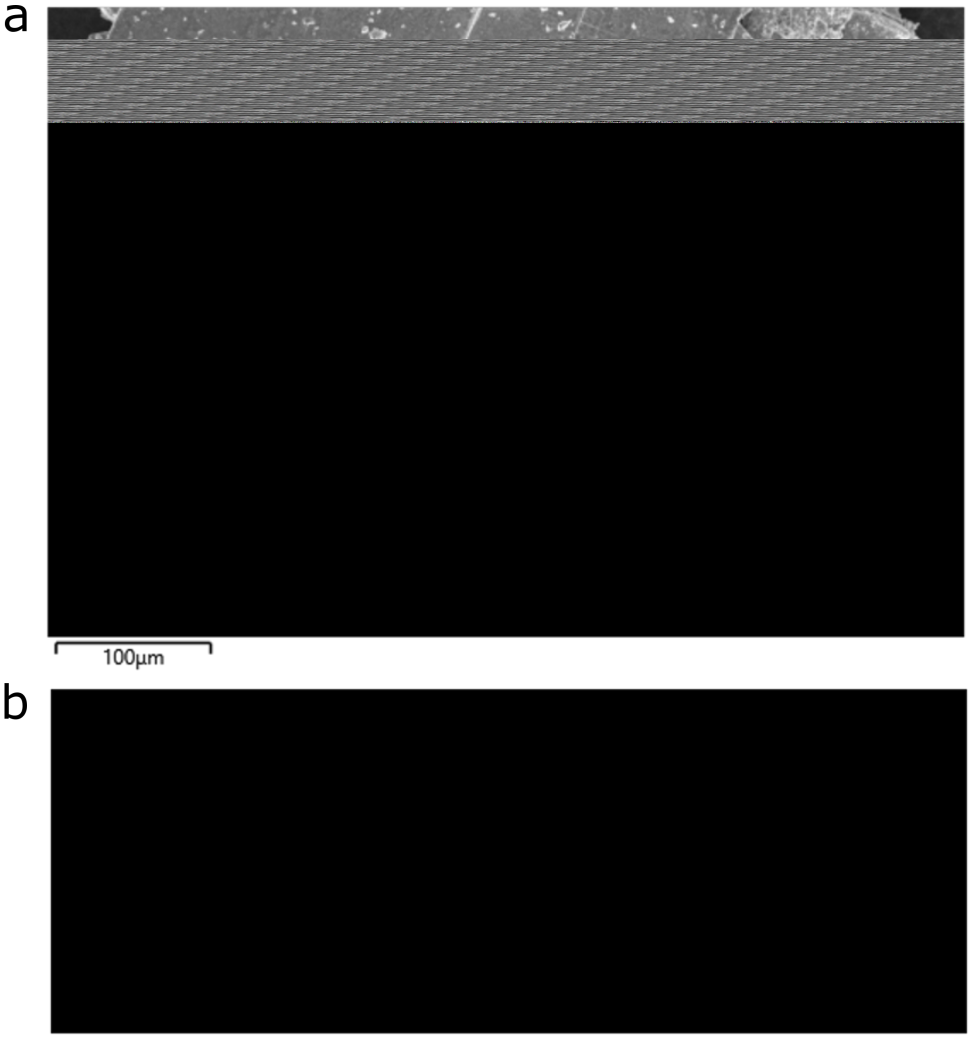

Fig. S2.: **SEM and EDX on $K_{0.5}WS_2$.** A SEM image (a) and an EDS spectrum (b) of a selected point on pristine $K_{0.5}WS_2$.

The chemical ratio of K in the pristine compound is determined by the EDS spectrum as shown in Fig. S2b. Therefore, the K is constrained to 0.5 per formula unit in the nominal composition shown in Table S1. The presence of oxygen in the EDS spectrum is attributed to the short period of air exposure while transferring the sample into the SEM chamber.

Table. S1.: Single crystal XRD data and structure refinement for $K_{0.33}WS_2$ at 299 K

Empirical formula	$K_{0.33}WS_2$
Nominal composition	$K_{0.5}WS_2$
Crystal system	Monoclinic
Space group	*C*2/*m*
Formula weight	521.94
Density (calculated)	5.846 g/cm^3
a (Å)	16.147(4)
b (Å)	3.2589(6)
c (Å)	5.6849(10)
β (°)	98.634(13)
Volume (Å^3)	295.76(10)
Z	2
Temperature (K)	299(1)
F(000)	449
Θ (°)	2.552 – 24.983
Crystal size (mm^3)	0.12 $\times$ 0.066 $\times$ 0.054
Absorption coefficient (mm^{-1})	40.592
R_{int}	0.0968
Refinement method	Full-matrix least-squares on F^2
Final R indices (R_{obs}/wR_{obs})	0.0320/0.0937
R indices (all data) (R_{all}/wR_{all})	0.0347/0.0945
Goodness-of-fit	1.167
Largest diff. peak and hole (e·Å^{-3})	1.828 and -2.406
The Cambridge Crystallographic Data Centre (CCDC) deposition number	2170254

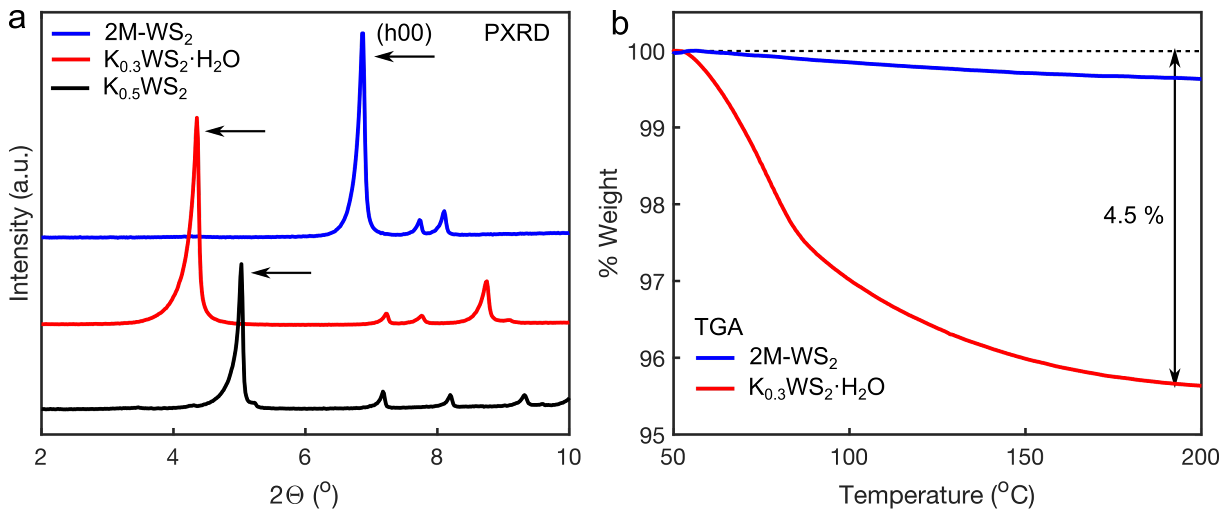

Fig. S3.: **Crystalline Water in partially deintercalaterd $K_{0.5}WS_2$.** (a) Low angle powder X-ray diffraction of fully K-deintercalated 2M-WS_2, partially K-deintercalated $K_{0.3}WS_2{\cdot}H_2O$, and the pristine $K_{0.5}WS_2$ to show the shift of (h00) peaks (X-ray: Mo $K_{\alpha 1}$). (b) Thermogravimetric analysis of fully K-deintercalated 2M-WS_2 and partially K-deintercalated $K_{0.3}WS_2{\cdot}H_2O$, showing the presence of crystal water in the partially deintercalated sample.

The partially K-deintercalated $K_{0.3}WS_2{\cdot}H_2O$ was obtained by shaking the pristine $K_{0.5}WS_2$ in diluted acid for an hour. We want to point out that shaking the pristine $K_{0.5}WS_2$ crystals in acidic solutions or plain water will not lead to successful delamination or exfoliation. Adding sonication will lead to delamination in acidic solutions. Sonication in water does not result in full deintercalation or exfoliation.

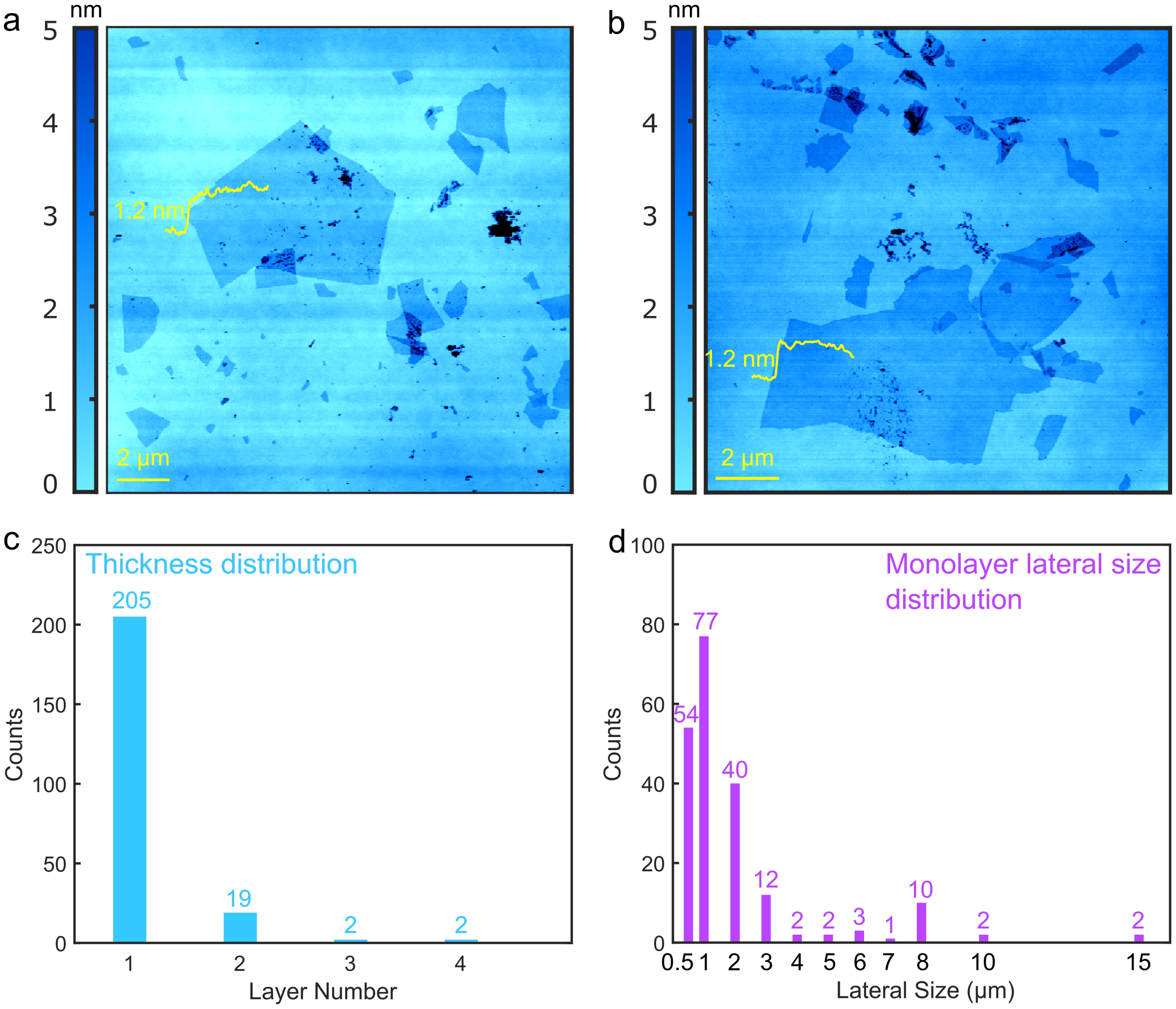

Fig. S4.: **Statistical analysis of the thickness and lateral size of 1T'-WS_2 nanosheets within the ink.** (a), (b) Two representative AFM images (out of 20 images taken in total) of dispersed 1T'-WS_2 monolayers on 285 SiO_2/Si wafers. (c) Counts of layer number. (d) Counts of the lateral size distribution. The longest lateral size of each sheet is used in the analysis.

Diluted 1T'-WS_2 nanosheets suspensions were dispersed on 285 nm SiO_2/Si wafers and scanned with AFM. Two selected scanned areas (out of 20 scanned areas) are shown in Fig. S4a and S4b. We scanned 7556 μm^2 with AFM to acquire a layer number distribution (Fig. S4c) and lateral size distribution (Fig. S4d) of the chemically exfoliated 1T'-WS_2 nanosheet suspension. The vast majority of nanosheets are monolayers. Among them, the median lateral

size is about 1 μm. However, large monolayers with lateral sizes up to 15 μms are not rare.

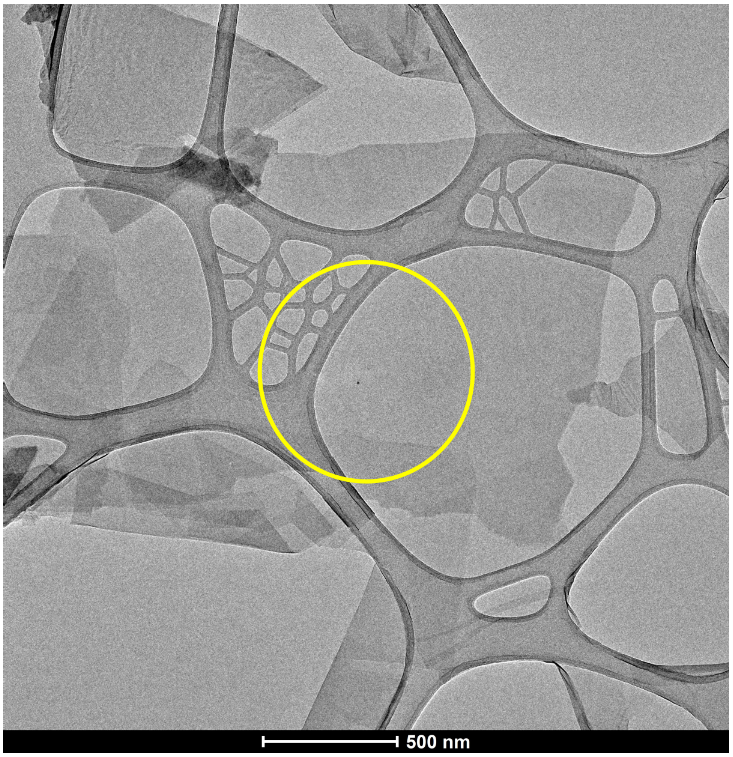

Fig. S5.: A TEM image of 1T'-WS_2 nanosheets. The yellow circle illustrates the selected area where the electron diffraction pattern shown in Fig. 1d is taken from.

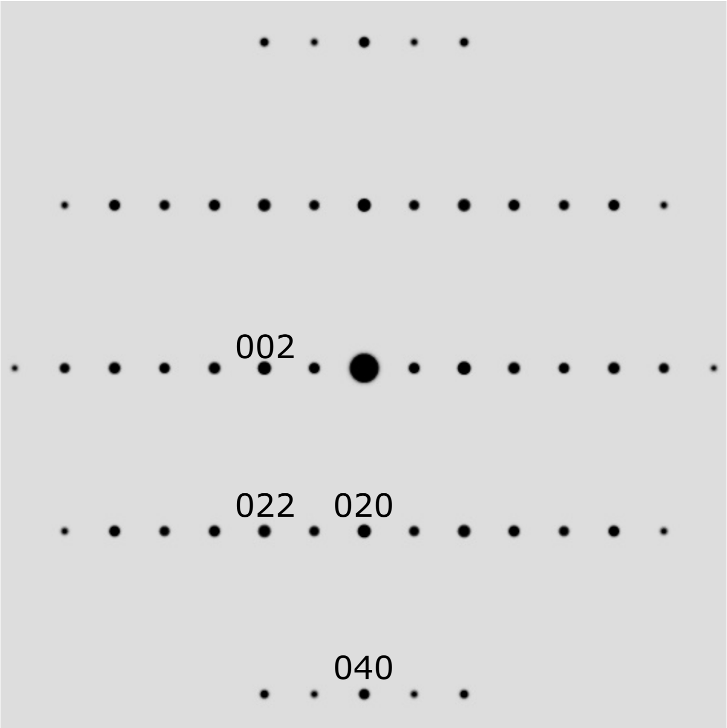

Fig. S6.: **Simulated electron diffraction pattern of 2M-WS_2.** The zone axis is <100>.

The electron diffraction pattern was simulated by the CrystalMaker software with 2M-WS_2's crystallographic information file.

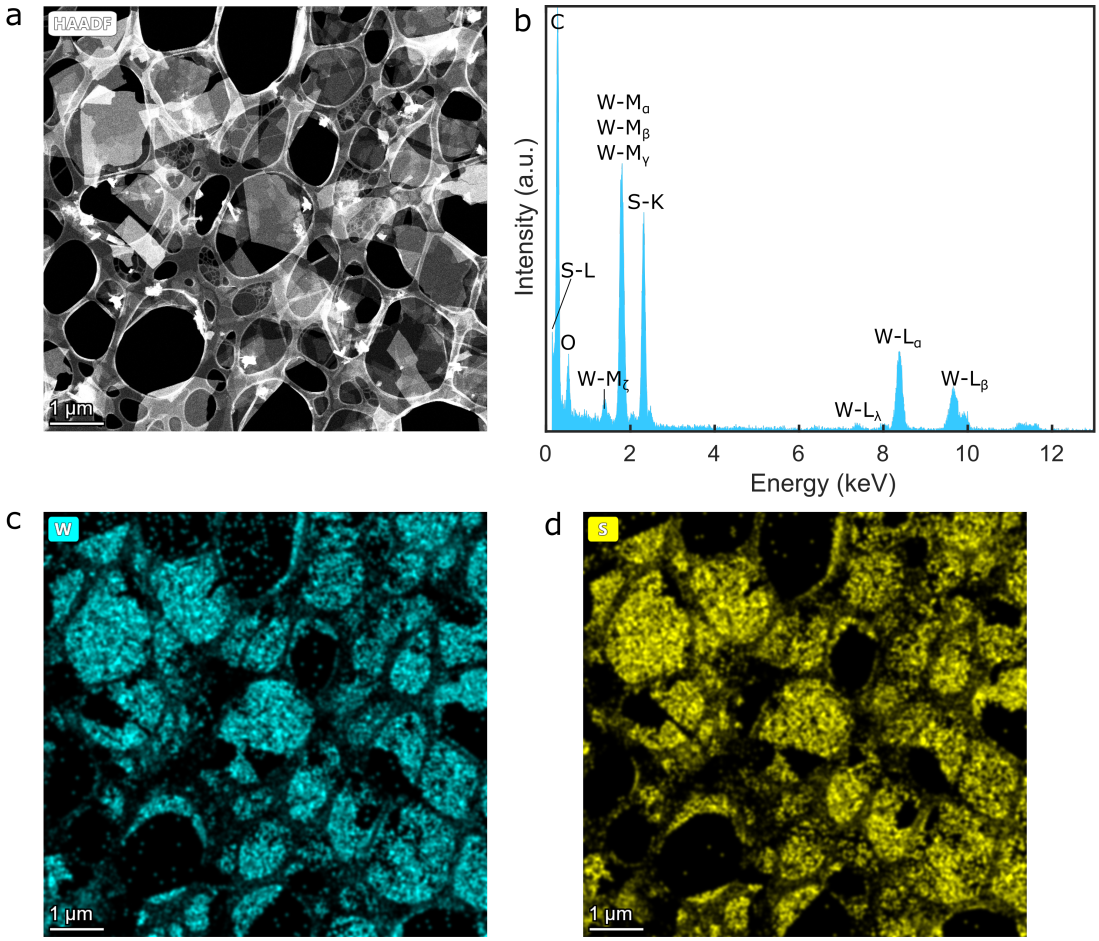

Fig. S7.: **Chemical composition of the 1T'-WS_2 nanosheets.** (a) A STEM image of 1T'-WS_2 nanosheets on top of a lacey carbon TEM grid. (b) The EDS spectrum of the entire region is shown in (a). (c) and (d) are EDS color maps of (a).

EDS analysis of the nanosheets was performed on an area of the TEM grid, as shown in Fig. S7a, where a large amount of 1T'-WS_2 nanosheets are stacked on top of each other to obtain enough signal for accurate analysis.

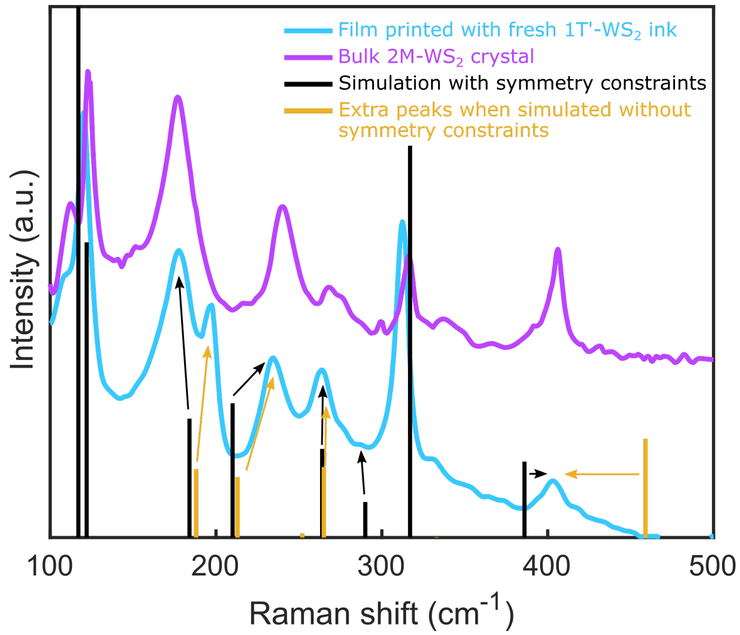

Fig. S8.: **Experimental vs. simulated Raman spectra.** Raman spectra of the freshly printed 1T'-WS_2 film (blue) and bulk 2M-WS_2 (purple) plotted against simulated Raman spectra of monolayer 1T'-WS_2. The simulated Raman modes with 3D symmetry (space group $P2_1/m$) constraints are plotted with black markers. The extra Raman modes generated by removing the symmetry constraints are plotted with light orange markers. The arrows indicate the assignment of simulated Raman modes to the experimental data.

The simulation of the monolayer was performed on a monolayer model with three-dimensional boundary conditions, with and without symmetry constraints, to understand the origins of the extra peaks in the printed 1T'-WS_2's experimental data. With symmetry (space group $P2_1/m$) constraints, the spectrum looks similar to the bulk, but without symmetry constraints, the number of signals doubles. In the Raman spectrum of the printed 1T'-WS_2, the extra peak at 196 cm^{-1} and the broadening of peaks around 400 cm^{-1} in comparison with that of the bulk 2M-WS_2 fit well with the calculation results without symmetry constraints.

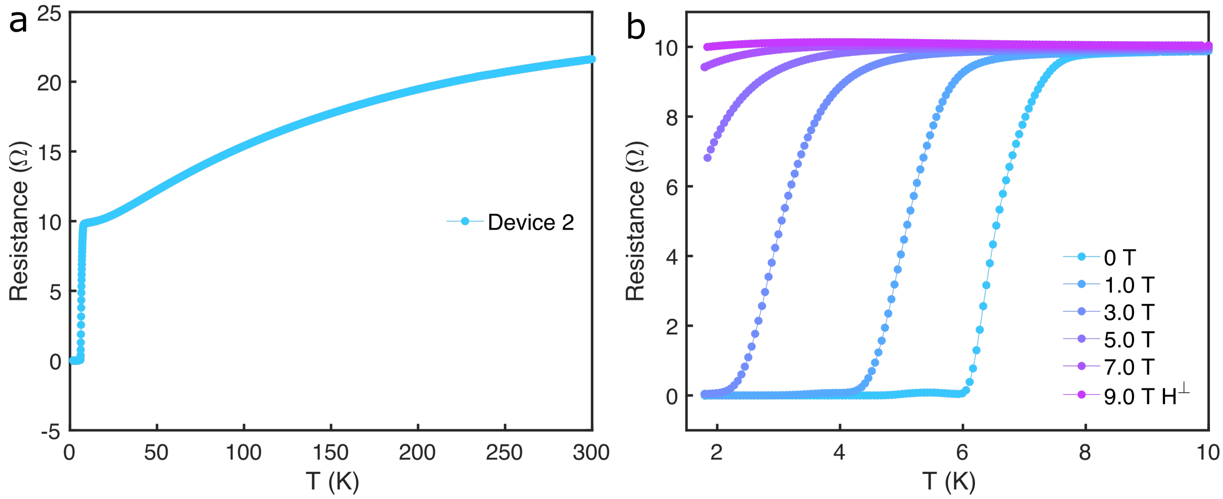

Fig. S9.: **Superconducting properties of a second device.** (a) Temperature (T) dependence of the resistance (R) for printed 1T'-WS_2 film device 2. (b) R-T data for device 2 at different magnetic fields, applied perpendicular to the device plane.

A different batch of 1T'-WS_2 nanosheet-ink was synthesized from a new batch of $K_{0.5}WS_2$. Then a second device (device 2) was printed from this new batch of nanosheet-ink. Transport measurements were performed on device 2 (Fig. S9), revealing very similar behavior to that of device 1. The device 2 has a RRR (R_{285K}/R_{8K}) of about 2.2.

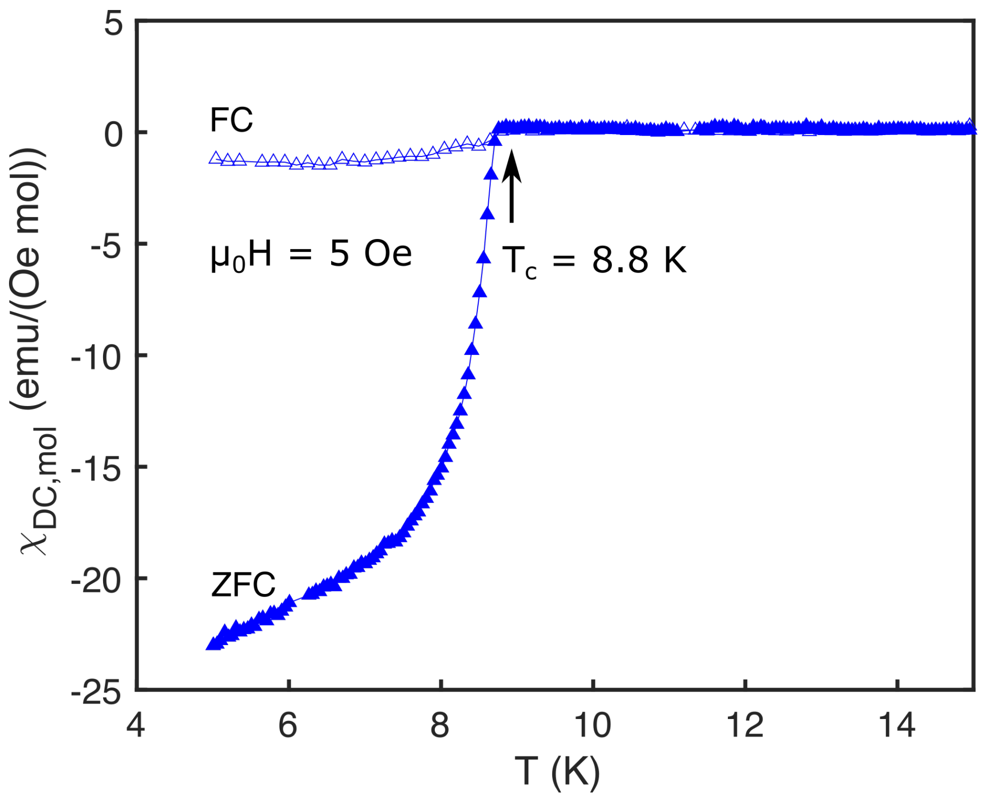

Fig. S10.: **Temperature-dependent magnetic susceptibility data of bulk 2M-WS_2.** Zero field cooling (ZFC) and field cooling (FC) data, with applied magnetic field of 5 Oe, are shown. The normal state to superconducting state transition appears at 8.8 K, identical to previous reports *(14)*.

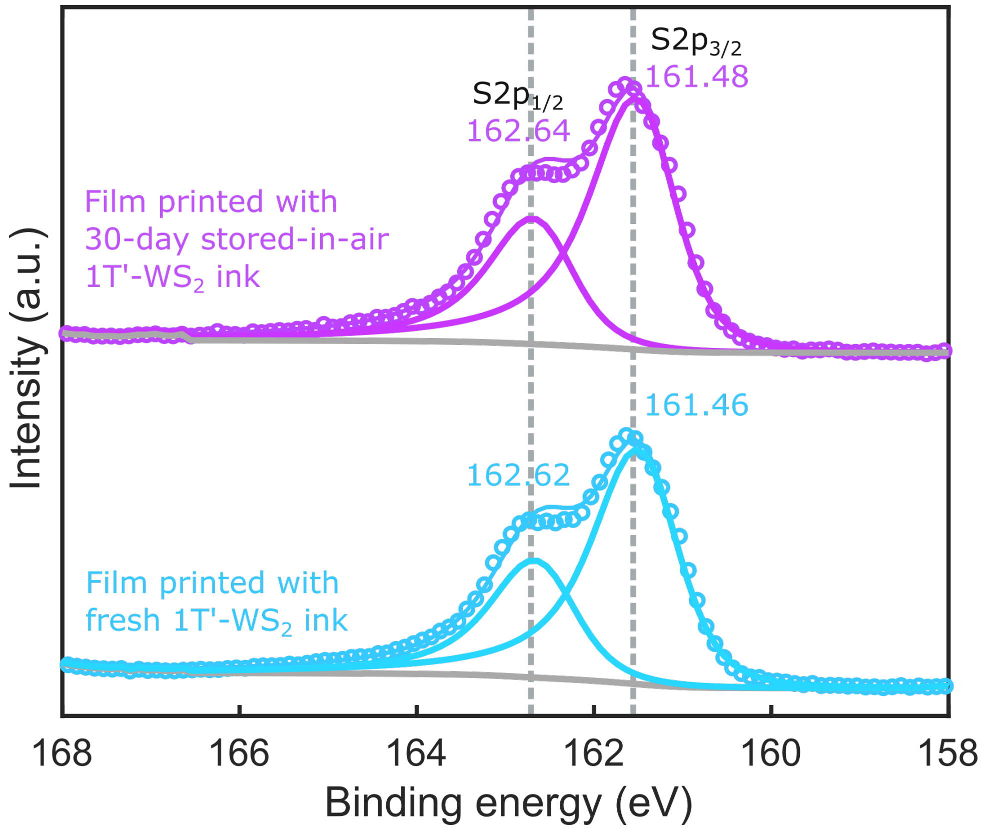

Fig. S11.: **Stability of the nanosheet-film analyzed with XPS.** S XPS spectra of the films printed with newly synthesized 1T'-WS_2 nanosheet-ink (blue) and the 1T'-WS_2 nanosheet-ink that is stored in air for a month (purple).

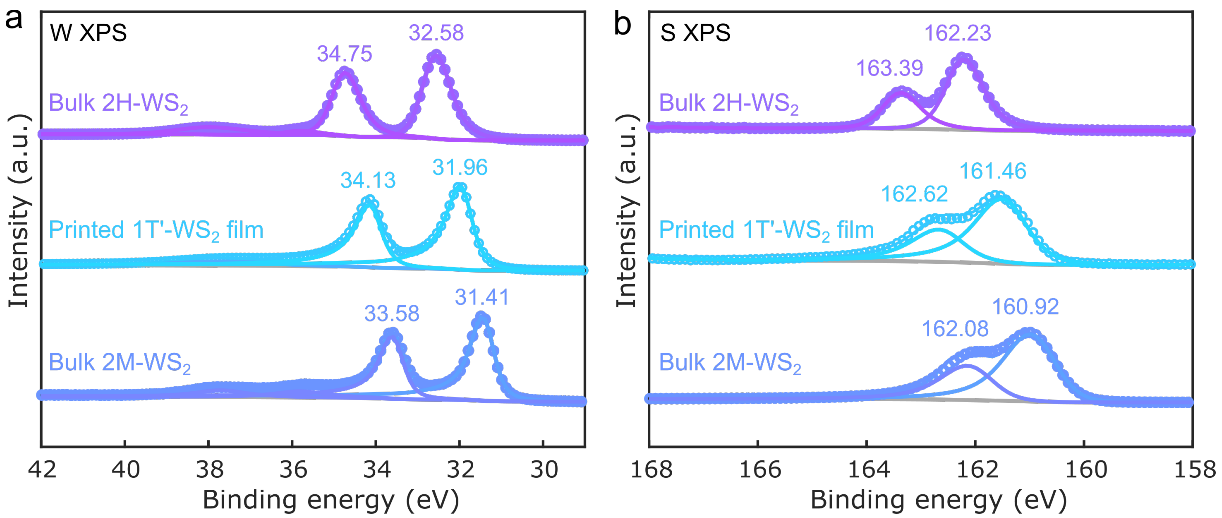

Fig. S12.: **Comparison of XPS spectra for bulk 2H-, 2M-, and printed 1T'-WS$_2$.** (a) W and (b) S XPS spectra of 2H-WS$_2$ bulk crystals, printed 1T'-WS$_2$ film, and the 2M-WS$_2$ bulk crystals.

2H-WS$_2$ bulk crystals were obtained by annealing 2M-WS$_2$ crystals at 400 °C. Their crystal structure was confirmed by PXRD. The binding energies of the W 4f XPS peaks of the printed 1T'-WS$_2$ films are higher than those of the bulk 1T'-WS$_2$ crystals, but lower than those of the bulk 2H-WS$_2$ crystals. We repeated the experiment with different batches of exfoliated nanosheets and found that the results are consistent. Note that our 1T'-WS$_2$ film was printed on a Cu tape rather than carbon tape, which is typically used for bulk crystals. This might affect the binding energies observed in XPS. Another cause of the difference might be related to the surface charge of the sheets.

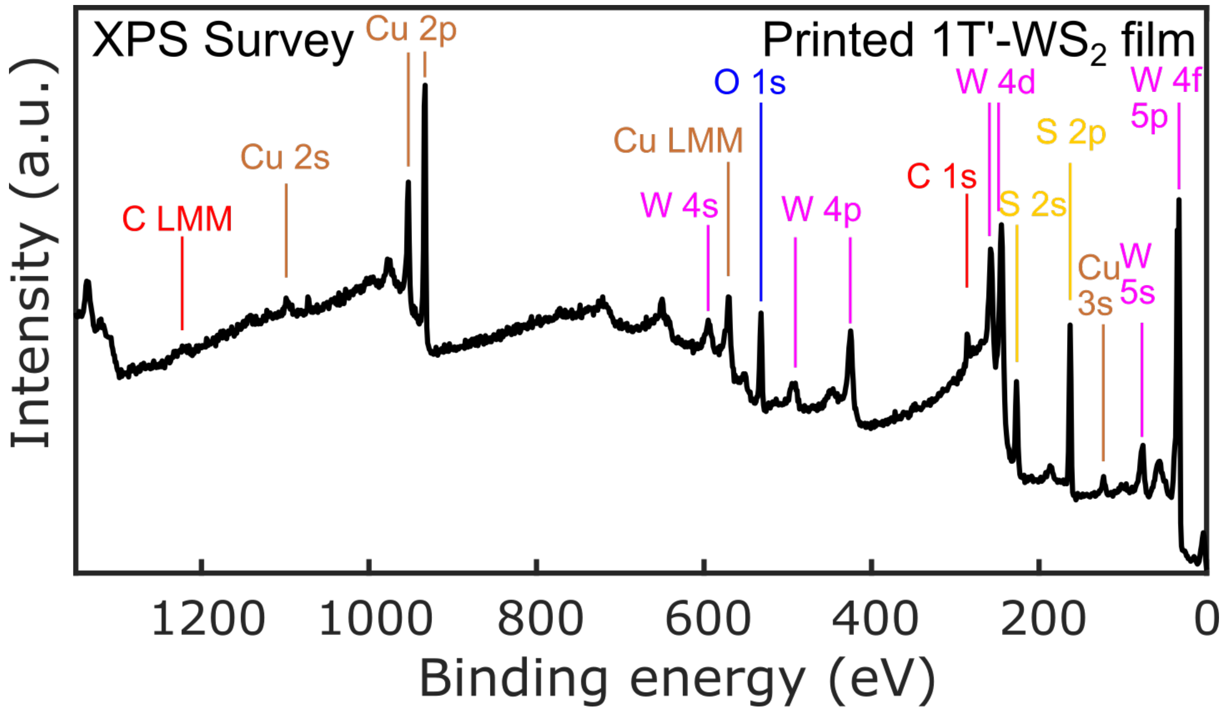

Fig. S13.: **XPS survey spectrum of a 1T'-WS_2 film printed on a Cu tape.**

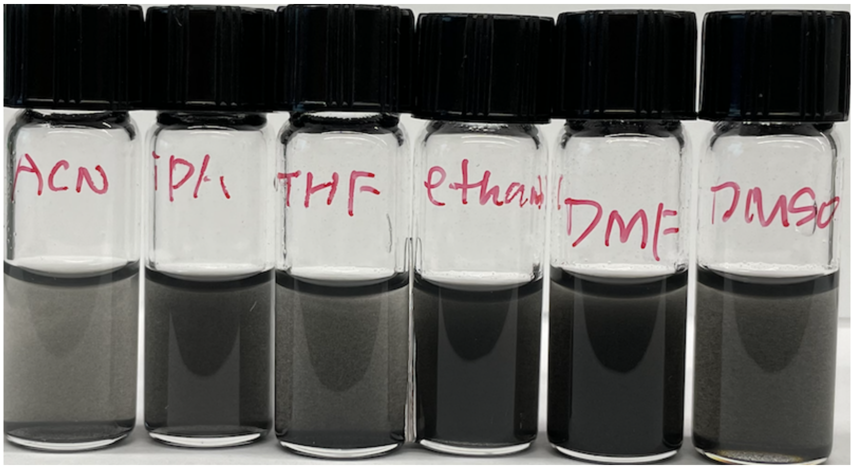

Fig. S14.: **1T'-WS_2 nanosheets redispersed in different solvents.** Solvents from left to right: acetonitrile (ACN), isopropanol (IPA), tetrahydrofuran (THF), ethanol, dimethylformamide (DMF), dimethyl sulfoxide (DMSO).

The 1T'-WS_2 nanosheet-powder was collected by centrifuging the aqueous nanosheet sus-

pension at 14000 rpm for 30 minutes to separate the solvent from the nanosheets. The nanosheets were dried under vacuum at room temperature. Then, the dried nanosheet-powder was sonicated in hexane, methanol, ethanol, IPA, acetone, acetonitrile, DMF, THF, and DMSO, separately, to test their dispersity in different solvents. The nanosheets do not disperse in hexane, methanol, and acetone. They can be redispersed in the solvents shown in Fig. S14. Ethanol and DMF give the best stability to the 1T'-WS_2 nanosheets among all tested solvents. However, water is the only tested solvent that can form a stable 1T'-WS_2 nanosheet suspension for several months.
